# Simulation as a Learning Tool in Cases of Aggression Against Healthcare Professionals

**DOI:** 10.1192/j.eurpsy.2026.11335

**Published:** 2026-07-31

**Authors:** P. Fernández De Romarategui Urresti, M. Sánchez López, C. B. Jose Manuel

**Affiliations:** Hospital Universitario De Bellvitge, Barcelona, Spain

## Abstract

**Introduction:**

Healthcare personnel are affected by various types of aggression during their professional activity.

In our center, incidents of violence have risen by 43% in the past year (6.7% involving physical assaults).

Protocols, legal support, and assistance from organizations and security staff must be complemented by training that equips personnel with the skills to manage and de-escalate conflict.

**Objectives:**

**Our objective is to adapt conventional aggression training to a practical, experiential format that enables professionals to apply their knowledge and skills in managing external violence.**

This aims to:

- Develop coping skills through realistic scenarios
- Identify and correct behavioral or attitudinal errors
- Enhance well-being through learning
- Create practical action sheets

**The methodology involves role-playing aggression cases followed by debriefing for reflection and analysis, complemented by pre- and post-training questionnaires to evaluate outcomes.**

**Methods:**

The methodology consists of simulating, through role-playing, cases of aggression and then conducting a debriefing session for reflection and analysis. In addition, questionnaires were administered at the beginning and end of the training to assess the degree to which objectives were achieved.

**Results:**

**After analyzing the initial and final questionnaires, we observed a clear improvement in students’ knowledge of how to act in cases of aggression.**

The graph shows the percentage of students who answered affirmatively before (PRE) and after (POST) the training.

**Main results:**

- *Knowledge of the first action to take in an aggression situation:* 100% after training vs. 41% initially.
- *Familiarity with de-escalation techniques:* 97% vs. 31%.
- *Ability to respond to verbal assault:* 100% vs. 48%.
- *Feeling prepared to manage an aggression situation in hospital settings:* 88% vs. 12%.
- *Confidence in applying calming techniques with aggressive patients or relatives:* 79.4% vs. 34%.

Overall, the course significantly improved participants’ preparedness, confidence, and ability to handle aggressive situations effectively.

**Conclusions:**

Experiential training has made it possible to increase the perceived sense of safety among staff who face these situations, as well as to achieve improvement and greater knowledge regarding:

- Actions to take in the event of verbal or physical aggression
- De-escalation (calming) techniques
- The influence of staff behavior when facing aggression
- Internal reporting procedures

**Disclosure of Interest:**

None Declared

